# Cocaine Poisoning

**Published:** 1891-09

**Authors:** 


COCAINE POISONING.—Doctor A. E. Roussel, in his "French Notes" for the Medical Digest department of the Times-Register, has translated from Le Bulletin Medical, M. Hallopean's history. An interesting case where an injection of one-seventh grain of hydrochlorate of cocaine in the neighborhood of a decayed tooth was followed by prolonged symptoms of acute cocainism. Which he accompanies with the following conclusions from his own personal observations.

1. A single hypodermic injection of cocaine may give rise not only to immediate symptoms of a severe type, but also to prolonged troubles of a painful character.

2. These symptoms much resemble those noticed shortly after an injection. They particularly consist of a persistent cephalaigia, accompanied by profound malaise, insomnia, and prostration, accompanied by vertigo, as well as cerebral excitation, which manifests itself by loquacity and great agitation.

3. Small doses of the medicament may be sufficient to cause the above.

4. Their duration may be of several months.

5. They are especially observed in patients of an excitable nervous system.

6. They may be attributed to an elective action of the poison on certain nervous centers.—*Alienist and Neurologist*,

